# Cumulative lifetime stressor exposure assessed by the STRAIN predicts economic ambiguity aversion

**DOI:** 10.1038/s41467-022-28530-2

**Published:** 2022-03-30

**Authors:** Candace M. Raio, Benjamin B. Lu, Michael Grubb, Grant S. Shields, George M. Slavich, Paul Glimcher

**Affiliations:** 1 Department of Psychiatry, New York University Grossman School of Medicine, New York, NY USA; 2 Neuroscience Institute, New York University Grossman School of Medicine, New York, NY USA; 3 Department of Psychology, Trinity College, Hartford, CT USA; 4 Department of Psychological Science, University of Arkansas, Fayetteville, AR USA; 5 Department of Psychiatry and Biobehavioral Sciences, University of California, Los Angeles, CA USA

**Keywords:** Human behaviour, Economics

## Abstract

Uncertainty is inherent in most decisions humans make. Economists distinguish between two types of decision-making under non-certain conditions: those involving risk (i.e., known outcome probabilities) and those that involve ambiguity (i.e., unknown outcome probabilities). Prior research has identified individual differences that explain risk preferences, but little is known about factors associated with ambiguity aversion. Here, we hypothesized that cumulative exposure to major psychosocial stressors over the lifespan might be one factor that predicts individuals’ ambiguity aversion. Across two studies (Study 1: *n* = 58, *M*_age_ = 25.7; Study 2: *n* = 188, *M*_age_ = 39.81), we used a comprehensive lifetime stressor exposure inventory (i.e., the Stress and Adversity Inventory for Adults, or STRAIN) and a standard economic approach to quantify risk and ambiguity preferences. Greater lifetime stressor exposure as measured by the STRAIN, particularly in early life, was associated with higher aversion to ambiguity but not risk preferences.

## Abstract (web summary)

Uncertainty is a factor in most decisions. Here the authors quantify tolerance for two forms of economic uncertainty—risk and ambiguity—and show that greater lifetime stressor exposure (as assessed by a comprehensive lifetime stressor exposure inventory) was associated with higher aversion to decisions involving ambiguity, but not risk.

## Introduction

The decisions that humans make in daily life often involve choosing amongst outcomes that are not certain. From simple choices about what to eat or wear to potentially serious health or financial decisions that can significantly impact our lives, the consequences of our choices can rarely be predicted with absolute certainty. Economists have long distinguished between two classes of non-certain outcomes: those in which the probability of an outcome is known (risky) and those in which the probability of an outcome is unknown (ambiguous)^1,2^. An example of a risky outcome might be choosing a poker chip from a bag that is known to contain 30 blue chips and 70 red chips. In this scenario, the odds of winning and losing are explicitly known in advance. An example of an ambiguous outcome, in turn, might be choosing from this same bag when the number of blue and red chips is completely unknown; without any additional knowledge, it is not possible to estimate the probability of getting a particular chip. Extensive evidence suggests that individuals vary significantly in their tolerance for both risk and ambiguity, which have been referred to as risk and ambiguity preferences. Perhaps surprisingly, these two tolerances are only weakly^3–5^ or not at all^6–8^ correlated.

Understanding the factors that shape risk and ambiguity preferences in individuals and across the lifespan has become a major interdisciplinary focus for researchers who recognize the tremendous impact that personal choices can have on interpersonal, financial, health, and legal outcomes. Although substantial progress has been made with respect to identifying individual differences that explain risk preferences^7,9,10^, far less is known about the psychosocial factors that shape ambiguity preferences, despite some economic^7,11,12^ and neuroscience^6,7,13^ research that has focused on this issue. In this report, we demonstrate across two studies that cumulative exposure to stressors over the lifespan appears to be selectively associated with peoples’ tolerance for ambiguity, but not risk, thus providing new insight into how life stress may contribute to decision-making under uncertainty.

Substantial research in economics has demonstrated that older individuals tend to be more risk averse than their younger counterparts. It has been unclear, however, whether this finding reflects a cohort effect or a natural feature of aging. Prior research^10^ has shown that this age-related change in risk aversion stems at least in part from reduced gray matter volume in the right posterior parietal cortex, a region that has been previously shown to modulate risk preferences in young, healthy adults. The fact that the cerebral cortex thins as we age^14^, and that thinning of the specific area previously associated with risk attitudes accounts for idiosyncratic risk preferences—whereas age has no effect on risk attitude once cortical volume is controlled for^10^—argues strongly for the possibility that age-related phenomena alter risk attitudes. In contrast, neither age nor cortical thickness appear to be related to ambiguity preferences^10^, which is consistent with the view that ambiguity preferences are phenomenologically distinct from risk preferences^5,7,8,10,15,16^. Although tolerance for risk and ambiguity are distinct, the factors that explain ambiguity aversion are less clear^16^.

Theoretical accounts of human stress and cognition suggest that stressor exposure may play a key role in shaping how people behave in ambiguous decision contexts^17–19^. For example, a large body of psychological research points to psychosocial stressor exposure as modulating how individuals appraise ambiguity. Threat or stressor exposure^20–22^ and negative affect^23^ have been associated with increased negative appraisals of ambiguous stimuli. Furthermore, individuals exhibiting higher trait anxiety have a greater tendency to rate ambiguous stimuli as negative or aversive^24–27^. Similar findings have been reported for predictable versus unpredictable threats, with the latter eliciting greater physiological arousal^28,29^, amygdala responsivity^30^, and avoidance behavior^25^, especially for individuals with greater exposure to trauma and adversity^31^.

Although this work has mainly focused on how proximal effects of stress or anxiety affect ambiguity appraisals, research stemming from the early life stress literature has long argued that cognitive responses to uncertainty can depend greatly on an individual’s past experience with trauma and adversity, pointing to more persistent effects of stressor exposure on behavior^31^. This work suggests that experiencing adversity over time may render future evaluations of ambiguous outcomes more negative. These cognitive biases are thought to arise because stressors signal the presence of threats in the environment^25^. Although these biases may be adaptive by helping ensure safety and survival in dangerous or volatile environments^32,33^, they can lead to overly pessimistic appraisals and avoidance of situations involving uncertainty^25^. This line of reasoning naturally leads to the hypothesis that exposure to stressors over the lifetime may predict greater aversion to decisions involving uncertainty. Furthermore, it suggests that this association may be selective to decisions for which outcomes are unknown (ambiguous) since these decisions require individuals to rely on their inferences and subjective estimates regarding the likelihood of a potential outcome occurring. This is in contrast to risky choices, where the likelihood of potential outcomes occurring is explicitly known.

Despite the possibility of this stress-cognition link, we are not aware of any studies that have directly examined how cumulative lifetime stressor exposure relates to uncertainty preferences, perhaps given the difficulty associated with systematically and comprehensively assessing stressors occurring over the entire lifespan^34–36^. Here, we address this issue by investigating this association using a newly developed, comprehensive, interview-based system that quantifies the major stressors an individual might have experienced over the lifetime^34–36^. We coupled this comprehensive assessment of lifetime stressor exposure with a widely-used and well-validated experimental economic decision-making paradigm^5,7,10^ that formally dissociates individuals’ economic preferences regarding risk and ambiguity. Based on the literature reviewed above, we hypothesized that greater lifetime stressor exposure—and early life stress in particular—would be related to a lower tolerance for ambiguity, but not risk.

**Fig. 1:**
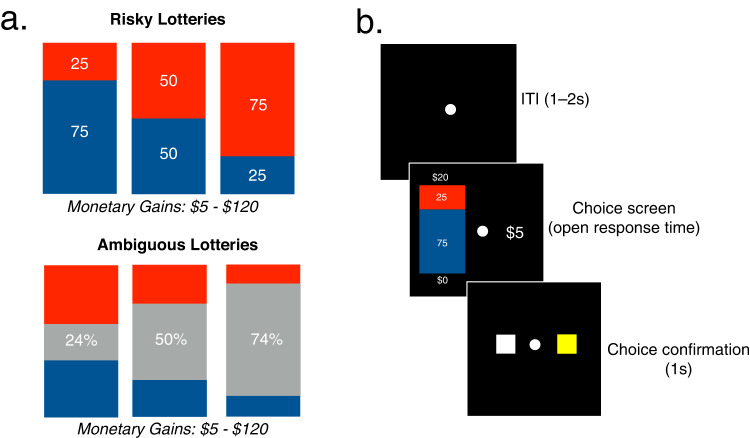
Task design. **a** All risky (top) and ambiguous (bottom) lottery bags used in the experiment. **b** Example trial sequence. Here, the participant chose between receiving $5 for sure or a lottery with a 25% chance of winning $20 and a 75% of winning $0.

To test this hypothesis, we conducted two studies in which healthy adult participants (Study 1: *n* = 58, 35 women, M_age_ = 25.7 ± 7.2 years, range 18–56; Study 2: *n* = 188, 82 women, M_age_ = 39.81 ± 12.14 years, range 19–73) made 240 decisions (Method) between a certain gain of $5 and playing a lottery in which the monetary amount to be won (20 values: $5–$120) and the probability of winning (0.25, 0.50, or 0.75) was explicitly known (risky lottery), or was partially unknown (ambiguous lottery). Ambiguity was manipulated by occluding a proportion (0.24, 0.50, or 0.74) of the winning probabilities from the lottery option, rendering the probabilities of winning the lottery partially unknown (Fig. 1a). This economic preference methodology is a well-validated decision-making task that independently quantifies risk and ambiguity preferences (Method; refs. ^5,7,10,15^). After completing all 240 decisions (Fig. 1b), participants completed the lifetime stressor exposure inventory (see below). Finally, one trial from the economic decision task was randomly selected and the monetary result of that lottery choice was compensated to the participant.

We quantified individuals’ attitudes toward risk and ambiguity by calculating the proportion of times participants chose the risky lottery over a certain option ($5) using a standard and well-validated choice set. This provided an estimate of participants’ risk attitudes with a higher or lower proportion of lottery choices indicating relative (rank order) risk tolerance among the participants^5,7,10,15^. Similarly, we calculated the proportion of trials for which participants chose the ambiguous lottery choices as a model-free (ordinal) index of ambiguity attitudes. Because ambiguous lotteries incorporate risk as well as ambiguity, the proportion of ambiguous lottery choices for each participant was first ‘risk-corrected’ by subtracting the proportion of risky lottery choices. This correction was applied to control for any influence of risk attitude in ambiguity attitude estimates. (For more details on this approach to measuring risk and ambiguity, see reference^7^ and Method.)

To quantify participants’ cumulative lifetime exposure to stress, we used the well-validated Stress and Adversity Inventory for Adults (STRAIN^36^). At the heart of the STRAIN is a detailed interview that quantifies an individual’s exposure to 55 different types of major acute and chronic stressors that may have occurred over the lifetime. The interview covers stressors occurring across 12 major life domains, including housing, education, work, health, marital/partner, reproduction, financial, legal/crime, life-threatening situations, etc. The interview also yields separate scores for stressors possessing five distinct social-psychological characteristics: interpersonal loss, physical danger, humiliation, entrapment, and role change/disruption. For each stressor that participants endorse, they are asked a series of tailored follow-up questions using a branching logic that captures the stressor’s severity, frequency, timing of exposure, and duration. Examples of stressors captured by the STRAIN include the death of a loved one, being fired from a job, experiencing homelessness or severe financial strain, being abused, and caregiving for a relative with major health issues (see Method for additional details). To index each participant’s cumulative exposure to stressors over the lifetime, we calculated the two primary indices of lifetime stressor exposure produced by the STRAIN: (a) total lifetime stressor count and (b) total lifetime stressor severity.

## Results

### Cumulative lifetime stressor exposure

Participants in Study 1 experienced an average of 14.55 stressors over the lifespan (*SD* = 10.70; range = 1–53; possible range: 0–166), with an average total lifetime severity score of 35.22 (*SD* = 26.75; range, 3–155; possible range: 0–265). This corresponds to an average severity rating of ‘moderately’ stressful for each stressor experienced.

### Choice behavior as a function of uncertainty type

**Fig. 2:**
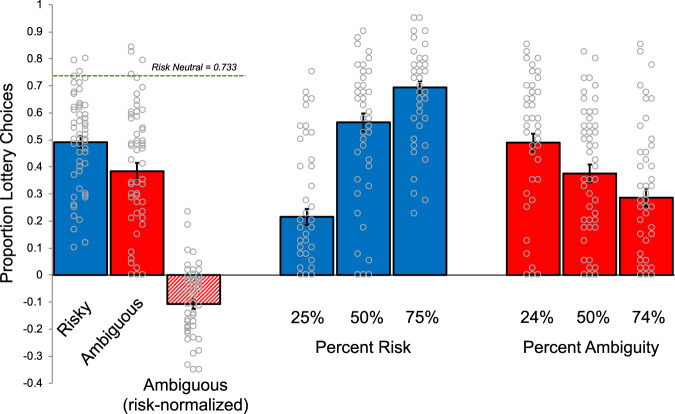
Lottery Choice Results (Study 1). Participants (*n* = 58) demonstrated risk (left, blue) and ambiguity aversion (left, red), even when accounting for risk (left, red/white diagonal). Further, participants chose the lottery option more as the probability of winning increased (blue, right) and less as the proportion of ambiguity increased (red, right). Overall, ambiguity was perceived as more aversive than risk. Errors bars indicate SE.

The proportion of trials for which participants chose the lottery option, averaged across all levels of risk and ambiguity (left) and for each level of risk (25%, 50%, 75%) and ambiguity (24%, 50%, 74%) (right), is shown in Fig. 2. As expected, individuals were generally risk averse (i.e., they chose the risky lottery less often than a risk-neutral chooser) and chose risky lotteries more as the probability of winning increased (Fig. 2, blue). We next examined how ambiguity affected participants’ willingness to gamble. Note that despite the increasing levels of ambiguity imposed on these trials, all of these ambiguous lotteries still have an objective winning probability of 0.50 (refs. ^7,8^); therefore, an ambiguity-neutral chooser should view ambiguous lotteries the same as 50% risky lotteries, whereas an ambiguity-averse chooser would treat them as having less than a 50% chance of winning. As expected, participants were ambiguity averse (i.e., they choose ambiguous lotteries less often than 50% risky ones) and tended to avoid ambiguous lotteries as ambiguity levels increased (Fig. 2, red). A similar pattern of choice behavior was observed even after partitioning participants into higher versus lower lifetime stress exposure groups using a median split of total lifetime stressor count (Supplementary Fig. 1).

### Lifetime stressor exposure is related to decreased ambiguous choice

**Fig. 3:**
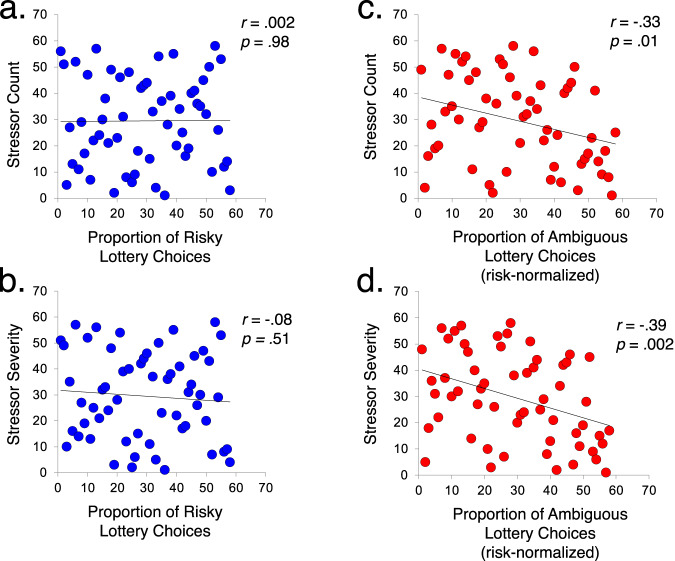
Effect of lifetime stressor exposure on estimates of risk and ambiguity tolerance (Study 1). Scatterplots depicting participants’ proportion of risky lottery choices (blue) plotted separately for total lifetime stressor **a** count and **b** severity, and proportion of ambiguous lottery choices (red) plotted separately for total lifetime stressor **c** count and **d** severity. A significant negative association emerged between both STRAIN indices and ambiguous lottery choices, indicating that greater lifetime stressor exposure was associated with less tolerance for ambiguity, while no such association was observed for risky lottery choices. Scatterplots depict non-parametric correlations (Spearman’s rho).

Given our a priori hypothesis that lifetime stressor exposure would be associated with ambiguity aversion, we first tested the association between participants’ lifetime stressor exposure scores and their proportion of ambiguous lottery choices. As depicted in Fig. 3, individuals who experienced more stressors over the life course were less likely to choose ambiguous lottery choices. Specifically, total lifetime stressor count and severity were both negatively associated with the proportion of (‘risk-corrected’) ambiguous lottery choices individuals were willing to accept (Spearman’s rho: total lifetime stressor count: *r*_s_ = −0.33, *p* = 0.01; total lifetime stressor severity: *r*_s_ = −0.39, *p* = 0.002). In contrast, no such relation emerged for risky lottery choices (total lifetime stressor count: *r*_s_ = 0.002, *p* = 0.98; total lifetime stressor severity: *r*_s_ = −0.08, *p* = 0.51). These findings suggest that greater lifetime stressor exposure and severity are selectively associated with a lower willingness to choose options for which the likelihood of potential outcomes is unknown.

**Fig. 4:**
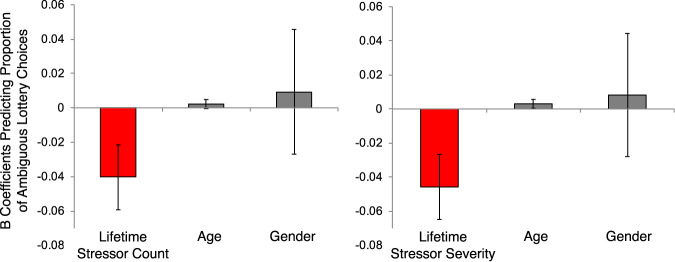
Multiple regression results for ambiguous lottery choice, lifetime stressor exposure, age, and gender (Study 1). Bars depicting B coefficients for proportion of (risk-corrected) ambiguous lottery choices controlling for age and gender. Lifetime stressor count and severity, but not age or gender, were significant predictors of ambiguous choice behavior. Data shown in bars are expressed as means; errors bars indicate SE. (*N* = 58).

Given that older participants have more years during which time they can experience stressors as compared to younger participants, coupled with the fact that gender differences often emerge in the human stress literature, we tested the robustness of these results by including age and gender as covariates using multiple linear regression. These analyses revealed that lifetime stressor count and severity—but not age or gender—were significant predictors of ambiguous lottery choice when these factors were included in the models (Fig. 4). Consistent with the results reported above, lifetime stressor exposure was still not a significant predictor of risky lottery choice rates while controlling for age and gender (Table 1) in this manner.

**Table 1:** Lifetime stressor exposure and ambiguous vs. risky lottery choice (Study 1).

	Ambiguous Lottery Choices *B (SE)*	*p value*	Risky Lottery Choices *B (SE)*	*p value*
Lifetime Stressor Count	−0.040 (0.019)	0.035*	−0.016 (0.024)	0.504
Age	0.002 (0.002)	0.388	0.005 (0.003)	0.104
Gender	0.009 (0.036)	0.803	0.019 (0.048)	0.684
Constant	−0.168 (0.069)	0.017*	0.341 (0.089)	0.0004**
	*B (SE)*	*p value*	*B (SE)*	*p value*
Lifetime Stressor Severity	−0.046 (0.019)	0.020*	−0.027 (0.025)	0.282
Age	0.003 (0.003)	0.256	0.006 (0.003)	0.072
Gender	0.008 (0.036)	0.821	0.016 (0.047)	0.728
Constant	−0.188 (0.070)	0.009**	0.323 (0.091)	0.0008**

Linear regression coefficients indicating the influence of lifetime stressor exposure on ambiguous vs. risky lottery choices (Study 1). Standard errors in parentheses. B coefficients significantly different from zero indicated by asterisks: *p < 0.05, **p < 0.01.

**Fig. 5:**
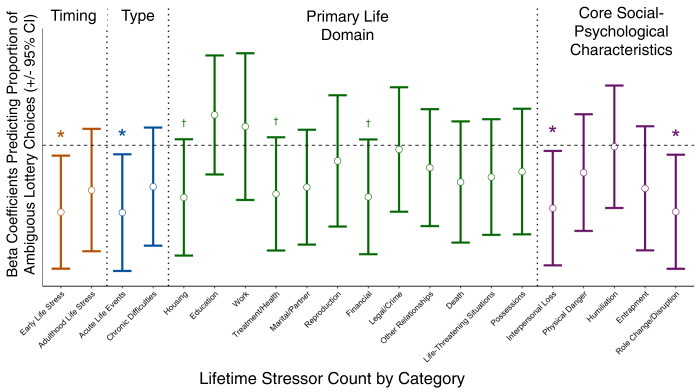
Stressor-specific effects on ambiguous lottery choice (Study 1). Standardized beta coefficients derived from linear regressions depicting the relation between lifetime stressor count and proportion of (risk-corrected) ambiguous lottery choices controlling for age and gender. Stressors on the y-axis are categorized by stressor timing (early life vs. adulthood), type (acute vs. chronic), primary life domain, and core social-psychosocial characteristic. Acute stressors (*p* = 0.024) and those experienced in early life (*p* = 0.021) were both predictive of ambiguous choice behavior, especially those marked by Interpersonal Loss (*p* = 0.032) or Role Change/Disruption (*p* = 0.023). Errors bars indicate 95% CI. Tests are uncorrected for multiple comparisons. *p < .05, ^†^*p* < .10. (*N* = 58).

Finally, to explore what features of lifetime stressor exposure relate to participants’ ambiguity tolerance, we examined how the timing of individuals’ stressor exposure (early life vs. adulthood) related to ambiguous lottery choice. As depicted in Fig. 5, only stressors occurring in early life (i.e., prior to age 18) were significantly associated with ambiguous choice behavior. In the present sample, therefore, stressors were not uniformly associated with participants’ ambiguity tolerance; rather, these effects differed based on when the stressors occurred, with those occurring early in life showing a significant association with ambiguity tolerance. Finally, an exploratory examination of the different social-psychological characteristics of lifetime stressors revealed that aversion to ambiguity was most strongly related to stressors involving Interpersonal Loss and Role Disruption (Fig. 5). Consistent with our primary hypothesis, a comparable exploratory analysis of the more classical risk tolerance yielded no significant associations with any lifetime stressor type or core social-psychological characteristic (see Supplementary Fig. 2).

### Study 2

To test the reproducibility of the association between cumulative lifetime stressor exposure and ambiguity aversion observed in Study 1, we conducted a replication study in which we used the same research rationale, study design, and analytic strategy as in the original study (Methods). This replication was performed in a larger independent sample derived from Amazon Mechanical Turk^37^ with the only difference being that we controlled for several additional potential confounds that could have influenced the results of the original study—specifically, socioeconomic status, IQ, and mental health status. These factors were included in the analysis because we reasoned that IQ and income could be related to the amount of stress individuals experience over the lifetime, and because lifetime stressor exposure—and early life stress in particular—has been shown to strongly predict increased risk for psychopathology. We controlled for these factors using an online version of the Raven’s Progressive Matrices^38^ (RPM) as a proxy for IQ, reported annual household income as an index of socioeconomic status, and the Kessler 6-Item Psychological Distress Inventory^39^ as a measure of psychological distress stemming especially from depression- and anxiety-related symptoms. Participants completed the same life stress measure (i.e., the STRAIN) and lottery task exactly as implemented in Study 1.

### Cumulative lifetime stressor exposure

Participants in Study 2 experienced an average of 14.78 stressors over the lifespan (*SD* = 12.05; range = 1–65; possible range: 0–166), with an average total lifetime severity score of 35.50 (*SD* = 27.64; range, 1–144; possible range: 0–265), which was comparable to that of Study 1.

### Lifetime stressor exposure is related to decreased ambiguous choice

Participants demonstrated risk- and ambiguity-aversion, and the proportion of lottery choices ordered as expected, with individuals generally choosing risky lotteries more often as the probability of winning increased, and ambiguous lotteries less often as the level of uncertainty increased (see Supplementary Fig. 3). Consistent with the results of Study 1, multiple linear regression including an estimate of IQ (RPM score), household income, and psychological distress (K-6 score)—along with our original covariates (i.e., age, gender)—revealed that lifetime stressor count and severity were both significantly associated with rates of ambiguous lottery choice but not risky lottery choice (Table 2). Age, gender, household income, RPM score, and psychological distress (K-6 score) were not significantly associated with ambiguous lottery choice (Table 2). Further confirming our original study results, these associations were significant for early life stressors but not for those experienced in adulthood, although we note that we observed a trend toward a significant association with adulthood stress (*p* = 0.059; Table 3 & Supplementary Fig. 4). Finally, we observed a similar pattern of results with respect to which specific social-psychological characteristics were associated with ambiguity aversion, with stressors involving Interpersonal Loss again emerging as a relevant type of stressor, though this time at a trend level (Supplementary Fig. 4). As in Study 1, risk tolerance was not significantly related to the exposure timing, type, or core social-psychological characteristics of the stressors experienced over the life course (Supplementary Fig. 5).

**Table 2:** Total lifetime stressor exposure and ambiguous vs. risky lottery choices (Study 2).

	Ambiguous Lottery Choice *B (SE)*	*p value*	Risky Lottery Choice *B (SE)*	*p value*
Lifetime Stressor Count	−0.025 (0.011)	0.024*	0.009 (0.017)	0.589
Age	0.001 (0.001)	0.084	0.000 (0.001)	0.959
Gender	−0.002 (0.020)	0.906	−0.043 (0.032)	0.177
IQ (RPM Score)	0.017 (0.042)	0.687	0.153 (0.066)	0.021*
SES (household income)	−0.011 (0.006)	0.064	0.005 (0.009)	0.595
Mental Health (K-6 score)	0.001 (0.002)	0.481	0.004 (0.003)	0.185
Constant	−0.149 (0.056)	0.010*	0.295 (0.089)	0.001**
	*B (SE)*	*p value*	*B (SE)*	*p value*
Lifetime Stressor Severity	−0.029 (0.011)	0.012*	0.003 (0.018)	0.869
Age	0.002 (0.001)	0.059	0.0001 (0.001)	0.896
Gender	−0.001 (0.020)	0.959	−0.043 (0.032)	0.183
IQ (RPM Score)	0.011 (0.041)	0.793	0.155 (0.066)	0.019*
SES (household income)	−0.011 (0.006)	0.068	0.005 (0.009)	0.637
Mental Health (K-6 score)	0.002 (0.002)	0.309	0.004 (0.003)	0.165
Constant	−0.161 (0.054)	0.006**	0.286 (0.091)	0.002**

Linear regression coefficients indicating the influence of lifetime stressor exposure on ambiguous vs. risky lottery choices (Study 2). Standard errors in parentheses. *RPM score* Raven’s Progressive Matrices, *SES* Socioeconomic Status, *K-6 score* Kessler 6-Item Psychological Distress Inventory. B coefficients significantly different from zero indicated by asterisks: *p < 0.05, **p < 0.01.

**Table 3:** Early life vs. adulthood total stressor exposure and ambiguous lottery choice (Study 2).

	Early Life Stressor Count *B (SE)*	*p value*	Adulthood Stressor Count *B (SE)*	*p value*
Ambiguous Lottery Choice	−0.022 (0.010)*	0.030*	−0.021 (0.011)	0.059
Age	0.001 (0.001)	0.277	0.001 (0.001)	0.083
Gender	−0.004 (0.020)	0.836	−0.003 (0.020)	0.885
IQ (RPM Score)	0.014 (0.042)	0.736	0.015 (0.042)	0.725
SES (household income)	−0.012 (0.006)	0.060	−0.011 (0.006)	0.083
Mental Health (K-6 score)	0.001 (0.002)	0.719	0.001 (0.002)	0.605
Constant	−0.111 (0.054)	0.042*	−0.145 (0.057)*	0.013
	Early Life Stressor Severity *B (SE)*	*p value*	Adulthood Stressor Severity *B (SE)*	*p value*
Ambiguous Lottery Choice	−0.023 (0.010)	0.024*	−0.023 (0.011)	0.049*
Age	0.001 (0.001)	0.285	0.002 (0.001)	0.066
Gender	−0.003 (0.020)	0.880	−0.002(0.020)	0.926
IQ (RPM Score)	0.013 (0.042)	0.747	0.009 (0.042)	0.829
SES (household income)	−0.012 (0.006)	0.060	−0.010 (0.006)	0.098
Mental Health (K-6 score)	0.001 (0.002)	0.680	0.001 (0.002)	0.462
Constant	−0.113 (0.054)	0.038*	−0.156 (0.059)	0.009**

Linear regression coefficients indicating the influence of early life vs. adulthood stressor exposure on ambiguous lottery choices (Study 2). Standard errors in parentheses. *RPM score* Raven’s Progressive Matrices, *SES* Socioeconomic Status, *K-6 score* Kessler 6-Item Psychological Distress Inventory. B coefficients significantly different from zero indicated by asterisks: *p < 0.05, **p < 0.01.

Standard errors in parentheses. *RPM score* Raven’s Progressive Matrices, *SES* Socioeconomic Status, *K-6 score* Kessler 6-Item Psychological Distress Inventory. B coefficients significantly different from zero indicated by asterisks: *p < 0.05.

## Discussion

Despite a growing body of work aimed at identifying factors that shape risk and ambiguity preferences in individuals and across the lifespan^5,6,8,10,13,15,16^, surprisingly little is known about the psychosocial factors that give rise to ambiguity aversion. This is especially striking given that choosers rarely have complete knowledge of the likelihood of real-world decision outcomes, suggesting that many decisions in daily life are made under conditions of (at least partial) ambiguity. Here, we examined the extent to which cumulative life stressor exposure may relate to economic preferences in two independent samples of healthy young adults. As hypothesized, across both studies, we found that the total number of major life stressors experienced across the lifespan and their cumulative lifetime severity were related to a lower willingness to choose ambiguous lottery options. These results were robust while controlling for age and gender in Study 1, and they replicated while controlling for an estimate of IQ (RPM score), annual household income, and mental health status (K-6 score) in Study 2. When we examined these associations by the different types of stressor exposure assessed by the STRAIN, we detected evidence of stressor-specific effects. Namely, across both studies, tolerance for ambiguity was associated with stressors occurring in early life (vs. adulthood) and, tentatively, with stressors involving Interpersonal Loss, such as the death of a parent. To our knowledge, these data are the first to directly document associations between lifetime stressor exposure and risk and ambiguity preferences.

A long tradition of theoretical and empirical work in economics has shown that individuals prefer known risk to unknown risk^1,40,41^ (for reviews, see^16,42,43^). Consistent with these classic demonstrations of ambiguity aversion, we found that individuals were more willing to accept risky lotteries than they were to accept ambiguous ones. Although evidence for ambiguity aversion is pervasive in the economic literature, the underlying source of it has been less clear. Since Ellsberg’s (^2^) classic demonstration (following Knight^1^) revealed that under uncertainty choosers behave in ways that violate normative models of decision-making, a number of different economic models have attempted to explain ambiguity aversion (for reviews, see^16,42,43^). One common feature of these theories is the central role that a chooser’s belief about unknown probabilities plays in shaping decisions under uncertainty. That is, when facing a risky lottery—for example, to win $100 with a 75% probability—a chooser has clear information on which to base their prediction of winning. In the absence of such information, however, one is left to infer the likelihood of acquiring this desired outcome. This suggests that past experiences where uncertainty yielded negative outcomes could potentially bias a person’s estimate of the likelihood of such an outcome occurring again in the future. Although risky decisions are still inherently uncertain, the fact that choosers routinely avoid these choices less frequently than they avoid ambiguous ones demonstrates that these forms of uncertainty are appraised differently. This distinction is especially important since much of the stress and decision-making literature conflates these two forms of uncertainty, rendering it difficult to distinguish whether stress exposure affects tolerance for risk or ambiguity. The present findings thus raise the question of whether greater life stress exposure, perhaps particularly early on in life, may shape the belief that choices with uncertain outcomes will result in a negative outcome, thus accounting for higher ambiguity aversion.

Consistent with this possibility, theoretical proposals have suggested that cumulative stress exposure over the life course—and especially in early life—may predict individuals’ decisions in contexts involving ambiguity^17,18^. Yet, the challenges associated with comprehensively measuring lifetime stressor exposure have rendered the association between these variables difficult to quantify. Here, we employed a well-validated and reliable lifetime stress exposure inventory to systematically measure stressors occurring over the life course^36^ and their relation to risk and ambiguity preferences. The comprehensive, yet adaptable nature of the STRAIN enabled us to objectively assess both the frequency and severity of a variety of different stressors that are known to affect cognition and health. Our economic decision-making task further enabled us to distinguish between two key forms of decision-making under non-certain conditions.

Our findings across two independent studies point to early life stress as being associated with ambiguity tolerance. One open question is why early life stress, but not adulthood stress, was significantly related to ambiguity aversion across both studies. This question points to two competing theoretical possibilities: One is that individuals who have experienced stress early in life may simply have the opportunity to accumulate more stressors over their lifetime (i.e., stress continuity), pointing to an additive effect of stress on ambiguity aversion. This account would best be supported if we had found that both early and adulthood stressors were related to ambiguity aversion. A second possibility stemming from the developmental literature suggests that since early life stressors are experienced during developmentally sensitive periods, this exposure modifies trajectories of cognitive and neural development to change the way uncertainty is appraised (irrespective of continued exposure to stressors later in life). This possibility is consistent with accounts of developmental programming, whereby stressful or traumatic experiences early in life can exert long-term effects on behavior. These changes are thought to reflect adaptations in cognitive and neural development that promote behavior that is optimally matched to one’s environment^17,18^. From this lens, increased ambiguity aversion could reflect an adaptive mechanism that emerges from early life experiences with stress. Although our data are largely consistent with the latter developmental programming account, we do note that adulthood stressor exposure was marginally related to ambiguity aversion in Study 2 (*p* = 0.059; Supplementary Fig. 4), making it difficult to definitively adjudicate between these two theoretical accounts based on the present two studies. Therefore, caution should be taken when interpreting a selective linkage between early life stressors and ambiguity aversion.

Why might a history of life stress exposure be related to aversion to ambiguity? Unlike conditions of risk where the likelihood of an event is fully described, conditions of ambiguity provide incomplete information about the likelihood of certain events occurring—that is, important information that could help a chooser decide in a way that better assures the desired outcome. While risky choices allow for accurate estimates about the probability of decision outcomes, ambiguous choices confer a higher degree of uncertainty, leaving decisions more vulnerable to subjective biases that are shaped by past experiences with stress and adversity^17,18,25,31,32^.

There are a number of potential mechanisms that may underlie these findings. First, greater life stressor exposure may be related to higher ambiguity aversion through associative learning mechanisms. Specifically, decisions involving ambiguity can instill greater perceptions of unpredictability and uncontrollability—two key features of stressful experiences^44^. Accordingly, recent research has shown that autonomic arousal selectively tracks perceptions of uncertainty (i.e., ambiguity^28^) and that the amplitude of these signals is greater when individuals choose ambiguous—relative to risky—gambles^8^. Therefore, perceptions of uncertainty and the process of evaluating uncertain options may be aversive to choosers if similar decision contexts led to negative outcomes in the past, especially during critical periods of development. Alternatively, life stressors may more directly shape the estimation process of calculating the likelihood of a particular outcome. This notion is consistent with a substantial literature showing that anxious or stressed individuals—especially those who have experienced early adversity—demonstrate a reliable and robust ‘negativity bias’ in the presence of ambiguous stimuli^21,23,26–28^ and perceive a higher likelihood of negative outcomes in hypothetical decisions involving uncertainty^45–48^. Past experiences with stress—particularly in early life—might thus confer a pessimistic view of unknown potential outcomes that manifests as higher ambiguity aversion. Thus, individuals who have experienced early life stress may generalize these experiences and predict an unfavorable outcome with a higher probability. Although risky decisions also involve some degree of uncertainty, choosers need not rely on subjective estimates to drive choice, perhaps explaining why we did not observe a similar association between life stress and risk tolerance. These cognitive mechanisms are consistent with a growing body of developmental research, which has shown that early life stress and adversity is associated with marked changes in the neural circuits involved in ambiguity processing, including the amygdala and ventromedial prefrontal cortex^31^, which play a critical role in associative learning, threat detection, emotion regulation^32^, and subjective value under risk and ambiguity^7^. Developmental acceleration of this circuitry has been shown across species and is thought to facilitate adequate coping with stressful environments^31^; therefore, it is possible that similar mechanisms may underlie our findings and generalize across the lifespan to decisions involving ambiguity.

Several limitations of this research should be noted. First, given the non-experimental nature of the study, the results are correlational and it is not possible to ascertain whether major stressors cause ambiguity aversion or, alternatively, whether people with a pre-existing predisposition to ambiguity aversion preferentially accumulate major stressors. Our data suggest that the former explanation may be more likely given that it was early life stressors that were more predictive across two studies, but additional research is needed to address this issue. Furthermore, when examining the social-psychological characteristics of the stressors associated with ambiguity aversion (see Fig. 5 and Supplementary Fig. 3), we found that it was stressors characterized by Interpersonal Loss that contributed to this association in Study 1 and tentatively in Study 2. Although these types of stressors seem unlikely to be caused by participants’ ambiguity tolerance, we cannot rule out the possibility that individuals suffer greater personal losses due in some way to their level of ambiguity aversion or to some common factor upstream of both stressors and ambiguity aversion.

Second, our assessment of early life stressor exposure used a cutoff age of <18. This cutoff is commonly used in the early life stress and trauma literature^38^ due to the fact that, from a developmental perspective, the types of stressors that individuals experience—and which have been shown to be most impactful—changes around age 18, as people move from experiencing stressors that primarily arise from being in a school and family environment (e.g., social rejection at school) to those that more commonly afflict independent adults (e.g., marriage difficulties). Nonetheless, it is possible that further segmenting early life stress into more narrow age intervals such as early versus later childhood^49^, or before versus after the pubertal transition, would reveal interesting differential associations with risk or ambiguity aversion that would be informative for understanding the development of these preferences under early stress exposure. Finally, although the STRAIN has been shown to be insensitive to negative mood and social desirability that can influence participants’ reporting of stressors^36,50,51^, it is possible that stressors could have been differentially recalled as a result of these or other factors, including individuals’ cognitive or resilience profiles. Therefore, we acknowledge that because life stressor exposure was retrospectively assessed, we cannot rule out possible reporting or disclosure biases.

Notwithstanding these limitations, the present findings address a long-standing question in economics regarding the psychosocial factors that may account for ambiguity aversion. Specifically, across two independent studies, we found that stressors occurring in early life—when individuals are developing formative statistical representations of their environment—are preferentially associated with a propensity to avoid ambiguous choice options. These findings differ from prior studies that have demonstrated a relation between more negative appraisals of ambiguity under acute stress^21,22^, which is known to recruit rapid physiological responses and promote immediate defensive responses to real or perceived threats^32^, or in anxiety, which reflects stable, trait-like tendencies for exaggerated anticipation of threats and the ensuing emotional consequences^24–27^. Our study is distinct in this regard as it examined cumulative exposure to stressful experiences across an individual’s lifetime and tested our hypothesis using an incentive-compatible (i.e., consequential) economic decision-making task that quantifies ambiguity aversion rather than relying on hypothetical assessments of ambiguity tolerance^45–48^. This approach is also distinct from existing emotional bias studies that have measured the frequency with which individuals endorse negative appraisals of ambiguous stimuli (e.g., facial expressions) using valence ratings^21–27^ as well as those that have measured physiological arousal signals when individuals appraise ambiguous stimuli^29–32^, often in the absence of explicit economic decision-making.

The present findings provide a potential experiential mechanism through which negative expectations about the environment may develop and shape decision preferences across the lifespan. Understanding the source of these preferences is highly relevant to everyday life, given that we rarely have complete information about the probability of different outcomes occurring for a wide variety of decisions that need to be made on a daily basis. Disentangling whether these ambiguity preferences are shaped by learning mechanisms that engender a bias toward negative predictions in ambiguous decisions contexts, or whether they reflect a broader aversion to ambiguity more generally is beyond the scope of this study but remains a relevant question to address in future research.

## Methods

### Participants

#### Study 1

Fifty-eight healthy young adult participants (35 women), aged 25.7 ± 7.4 years (range 18–56) participated in Study 1. Participants were recruited using flyers posted on and around the New York University (NYU) campus as well as electronic advertisements on NYU’s Department of Psychology website. All participants provided written informed consent. All research procedures were approved by NYU’s University Committee on Activities Involving Human Subjects. Participants were paid $15 per hour plus additional compensation based on the result of a randomly selected trial from the lottery task.

#### Study 2 (Replication study)

Participants (*n* = 210) for Study 2 were recruited on Amazon Mechanical Turk. Data from 6 participants failed to record and 18 participants were excluded for demonstrating a failure to understand task instructions based on choice errors, defined here as violations of ‘first-order stochastic dominance’ in greater than 15% of choices. The final sample thus consisted of 188 adult participants (82 women) aged 39.8 ± 12.14 years (range 19–73). Participants were compensated $10 for their time in addition to a potential bonus payment generated by a randomly selected trial from the lottery task. All participants provided informed consent and all experimental procedures were performed in accordance with approved protocols and regulations by the New York University Langone Health Institutional Review Board. The economic decision-making task and life stress measure (STRAIN) were both identical to those used in the original study. IQ scores were estimated using an online version of the Raven’s Progressive Matrices (RPM score), which is a test widely used in the psychology literature to measure abstract reasoning and non-verbal fluid intelligence^38^. Participants reported their annual household income range over the last year as an estimate of their socioeconomic status and their mental health status using the Kessler 6-Item Psychological Distress Inventory^39^, which is a self-report survey that measures psychological distress over the past month particularly related to anxiety and depression. All tests were two-tailed and considered statistically significant when *p* < 0.05.

### Stress and Adversity Inventory for Adults (STRAIN)

To acquire a comprehensive assessment of participants’ stressor exposure across the life course, participants completed the Stress and Adversity Inventory for Adults (STRAIN^36^). The STRAIN is an online stress assessment system that asks participants whether or not they have experienced 55 different major acute and chronic stressors (see https://www.strainsetup.com). Questions are presented serially and participants respond by clicking on the computer screen. For each stressor that is endorsed, participants are asked a series of follow-up questions pertaining to that stressor’s perceived severity, frequency, timing, and duration. For example, a participant may be asked “Have you ever been laid off or fired from a full-time job?”. If they respond “Yes”, then they are asked how many times this stressor occurred, how stressful or threatening it was (at its worst), what point in the participant’s life it occurred, and for how long the stressor was present. This enabled us to quantify the severity and frequency of stressors experienced across the lifespan, and to identify stressors experienced early in life (i.e., before age 18) versus adulthood, as well as those that were acute life events (i.e., those lasting a few days) versus chronic difficulties (i.e., those lasting several months or years). The stressors assessed by the STRAIN span 12 major life domains (e.g., housing, work, financial, marital/partner relationship, etc.) and 5 social-psychological characteristics (e.g., interpersonal loss, physical danger, humiliation, etc.). We calculated the two primary indices of lifetime stressor exposure produced by the STRAIN: (a) total lifetime stressor count (i.e., objective stressor exposure) and (b) total lifetime stressor severity (i.e., subjective stress experience), and further focused on the timing of the stressor exposure (i.e., early life vs. adulthood). STRAIN scores were z-transformed prior to performing linear regressions.

The STRAIN has the advantage of being broad in coverage, yet specific and quantifiable, as it is designed to measure the frequency and severity of discrete stressors that impact cognition and health. This is in contrast to other measures that assess overall perceived stress burden, which can be conflated with individuals’ cognitive biases or personality^34,35^. The STRAIN has high test-retest reliability (test-retest correlation of up to *r*_icc_ = 0.95 over one month; see ref. ^50^) and has been validated across a number of recent studies^36,50,51^. Finally, scores from the STRAIN have been shown to predict an array of affective and cognitive processes in healthy samples, including acute stress reactivity^52^, self-reported mental and physical health^53–56^, and stimulus-response memory^57^ and working-memory performance^58^.

### Economic decision-making task

We assessed economic preferences using a well-validated decision-making task that independently quantified risk and ambiguity preferences^5,7,8,10,15^. Participants first completed a brief training session that included visual and verbal explanations of the choice task, 40 practice trials and a brief comprehension quiz. During the choice task, participants made 240 decisions between a certain and uncertain option. The certain option was a 100% chance of winning $5 and was available on every trial. To independently assess participants’ tolerance for risk and ambiguity, the uncertain option was either a risky lottery—where the probability of winning (0.25, 0.50, or 0.75) was explicitly known—or an ambiguous lottery—where the probability of winning was partially unknown. All lotteries provided a chance to win $5 or more (20 monetary values: $5–$120) or a chance to win nothing.

Figure 1a presents the three possible risky and ambiguous lotteries that participants encountered during the task. Lotteries were portrayed on the screen using an image of a vertical bar that represented a bag of 100 poker chips. Participants were told that this bag contained both red and blue chips, and that the proportion of the blue and red color in the bar, as well as the corresponding numbers written on the colored areas, represented the probability of winning each lottery. For example, in Fig. 1b, a participant would choose between the certain payoff of $5 (right) or the depicted risky lottery option (left). In this example, 75% of the bar is colored blue and 25% is colored red, indicating that there are 75 blue chips and 25 red chips. This meant that participants have a 25% chance of winning $20 and a 75% chance of winning $0. To facilitate participants’ understanding, physical bags filled with the presented proportions of red and blue chips were available for participants to equate the act of playing a lottery with “drawing a chip” from the bag. We used three levels of winning probability (i.e., 0.25, 0.50, or 0.75) for risky lotteries.

For ambiguous lotteries, we occluded a portion of the colored areas representing probability using a gray bar of varying size. The size of the occluder indicated one of the three levels of ambiguity (i.e., 24%, 50%, 74%). For example, in the lower panel of Fig. 1a, the middle lottery option depicts 25% red and 25% blue. However, since the remaining 50% is occluded with the gray bar, then the probability of picking a blue chip could be anywhere between 25% (if all the chips behind the occluder are red) and 75% (if all the chips behind the occluder are blue). The probability of winning in these trials was therefore incompletely known (i.e., ambiguous). Importantly, we controlled the objective probability of winning ambiguous lotteries to always be 0.50. This enabled us to quantify individuals’ ambiguity aversion in addition to their risk aversion.

Each participant faced the same set of 240 choices presented in randomized order, each using an open response-window. Participants pressed one of two keys to indicate their choice, and this response was immediately confirmed by a 1-second presentation of the pressed button (Fig. 1b). A jittered 1–2 second inter-trial-interval followed, during which time a white fixation dot was presented. The screen position of each option (certain versus lottery) was counterbalanced across trials, as was the assignment of red and blue to the different monetary amounts in the lottery.

In total, there were 120 unique reward magnitude and reward probability combinations and each combination was presented twice for a total of 240 trials, divided into eight blocks. Choices were presented using PsychToolBox (Study 1) and on Amazon Mechanical Turk (Study 2). Participants made their choices using their keyboard. After completing all 240 decisions (Fig. 1b), participants completed the STRAIN. Finally, one trial from the economic decision task was randomly selected and the result of that lottery choice was realized in the form of bonus compensation.

### Reporting summary

Further information on experimental design is available in the Nature Research Reporting Summary linked to this paper.

## Supplementary information

Supplementary Information

Reporting summary

## Data Availability

The anonymized data generated in this study have been deposited in the Open Science Framework database (https://osf.io/qvku6/). Source data are provided alongside the figures in this article. Source data are provided with this paper.

## Source data

Source Data
