# Author Correction: Cumulative lifetime stressor exposure assessed by the STRAIN predicts economic ambiguity aversion

**DOI:** 10.1038/s41467-023-39380-x

**Published:** 2023-06-15

**Authors:** Candace M. Raio, Benjamin B. Lu, Michael Grubb, Grant S. Shields, George M. Slavich, Paul Glimcher

**Affiliations:** 1 Department of Psychiatry, New York University Grossman School of Medicine, New York, NY USA; 2 Neuroscience Institute, New York University Grossman School of Medicine, New York, NY USA; 3 Department of Psychology, Trinity College, Hartford, CT USA; 4 Department of Psychological Science, University of Arkansas, Fayetteville, AR USA; 5 Department of Psychiatry and Biobehavioral Sciences, University of California, Los Angeles, CA USA

**Keywords:** Human behaviour, Economics

Correction to: *Nature Communications* 10.1038/s41467-022-28530-2, published online 30 March 2022

The original version of this Article contained an error in Fig. 3, in which the axes labels were reversed. The label “Stressor Count” in Fig. 3a and 3c and “Stressor Severity” in Fig. 3b and 3d appeared on the x-axes, but were meant to appear on the y-axes. The labels “Proportion of Lottery Choice” in Fig. 3a and 3b and “Proportion of Ambiguous Lottery Choices (risk-normalized)” in Fig. 3c and 3d appeared on the y-axes but were meant to appear on the x-axes. This has been corrected in both the PDF and HTML versions of the Article.

