# Supplementary material for: Cumulative lifetime stressor exposure assessed by the STRAIN predicts economic ambiguity aversion
Source: Nat Commun. 2022 Mar 30;13:1686. doi: 10.1038/s41467-022-28530-2 (PMC8967930; doi:10.1038/s41467-022-28530-2)
Supplement: Supplementary file 1 — Supplementary Information [file 41467_2022_28530_MOESM1_ESM.pdf]

Supplementary Information

Cumulative lifetime stressor exposure assessed by the STRAIN predicts economic ambiguity aversion

Candace M. Raio[1*], Benjamin B. Lu[2], Michael Grubb[3], Grant S. Shields[4], George M. Slavich[5], and Paul Glimcher[2]

[1] Department of Psychiatry, New York University Grossman School of Medicine, New York, NY, USA; [2] Neuroscience Institute, New York University Grossman School of Medicine, New York, NY, USA; [3] Department of Psychology, Trinity College, Hartford, CT, USA; [4] Department of Psychological Science, University of Arkansas, AR, USA; [5] Department of Psychiatry and Biobehavioral Sciences, University of California, Los Angeles, CA, USA

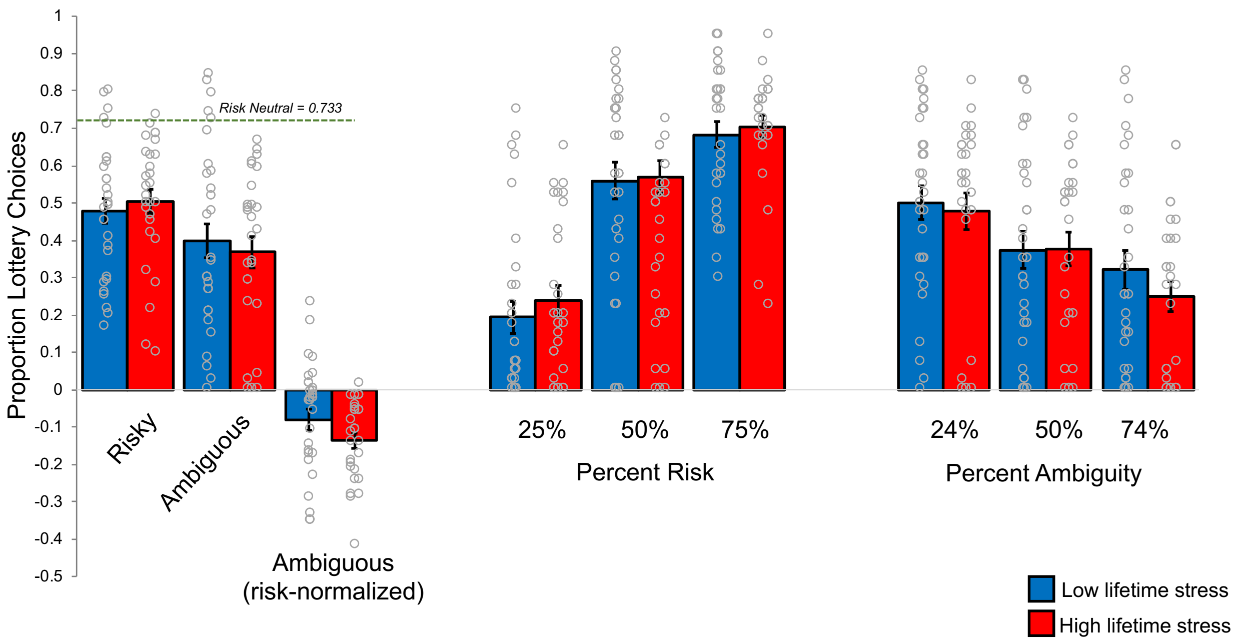

Supplementary Figure 1. Choice results as a function of participants' lifetime stressor exposure (Study 1). A median split of participants based on high (red) vs. low (blue) total lifetime stressor count as measured by the STRAIN. All participants (n=58) showed risk and ambiguity aversion with increased gambling as the probability of winning increased and decreased gambling as the level of ambiguity increased. Errors bars indicate SE.

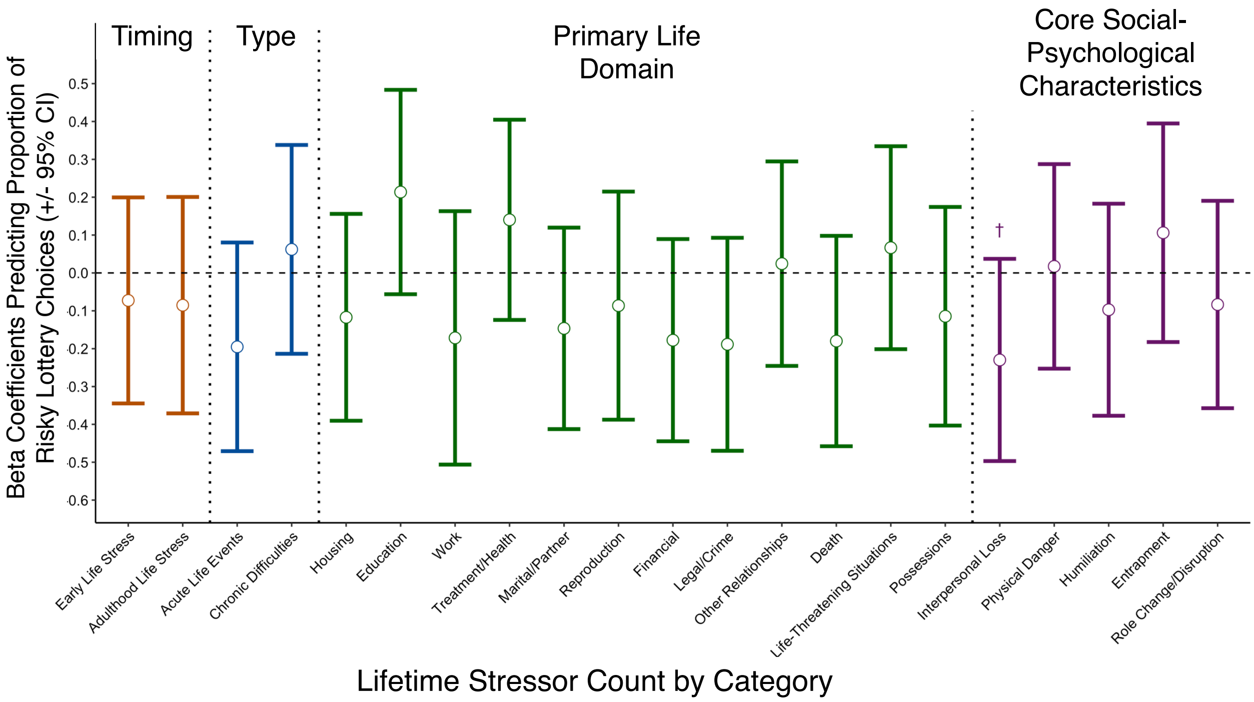

Supplementary Figure 2. Stressor-specific effects on risky lottery choice (Study 1, *n*=58). Standardized beta coefficients derived from linear regressions depicting the relation between lifetime stressor count and proportion of risky lottery choices controlling for age and gender. Stressors on the y-axis are categorized by stressor timing (early life vs. adulthood), type (acute vs. chronic), primary life domain, and core social-psychosocial characteristics. No stressors were significantly associated with risky choice behavior. Tests are uncorrected for multiple comparisons. Errors bars indicate +/- 95% CI. $^{\dagger}p < .10$

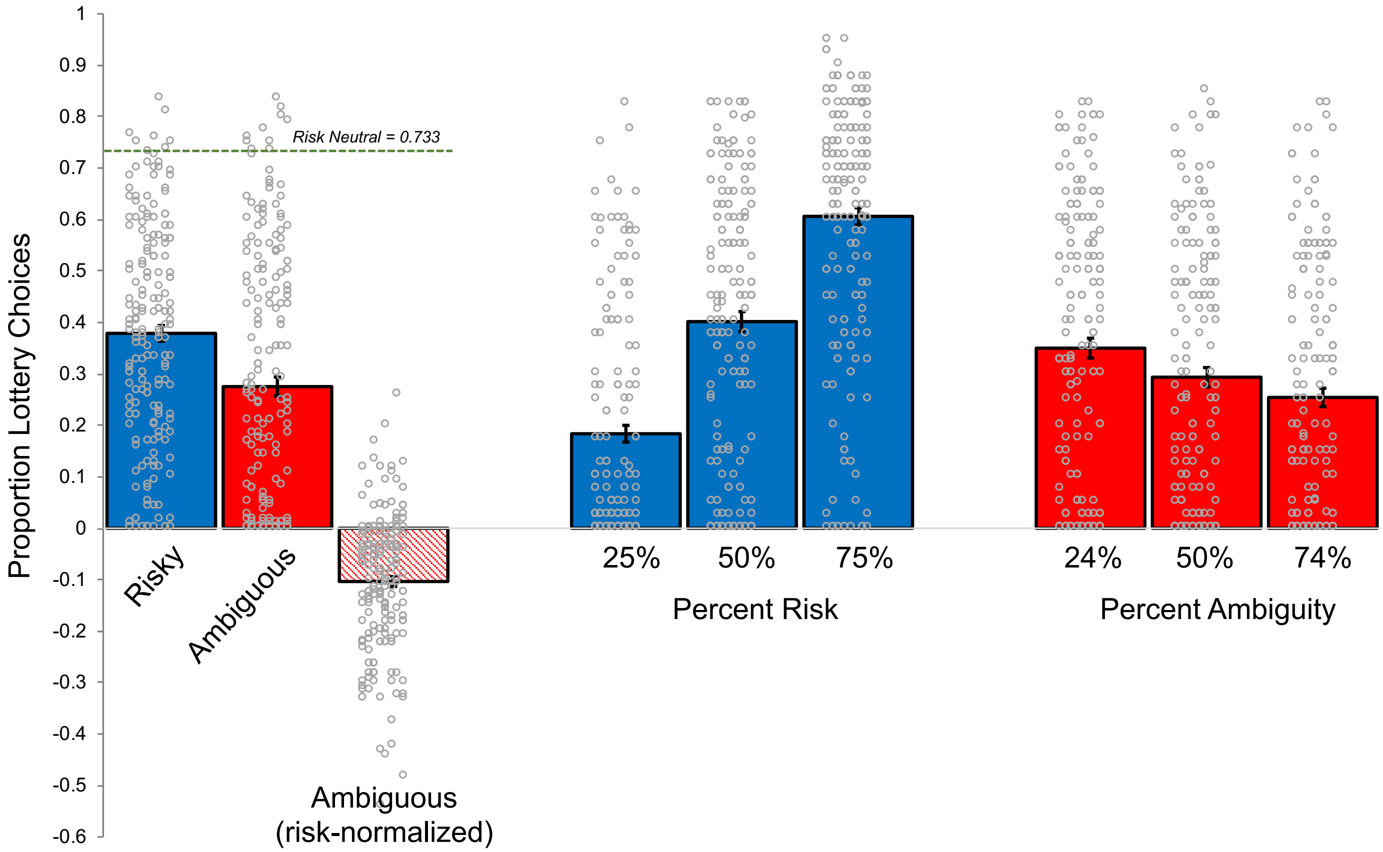

Supplementary Figure 3. Lottery Choice Results (Study 2). Participants (*n*=188) again demonstrated risk (left, blue) and ambiguity aversion (left, red), even when accounting for risk (left, red/white diagonal). Further, participants chose the lottery option more as the probability of winning increased (blue, right) and less as the proportion of ambiguity increased (red, right). Overall, ambiguity was perceived as more aversive than risk. Errors bars indicate SE.

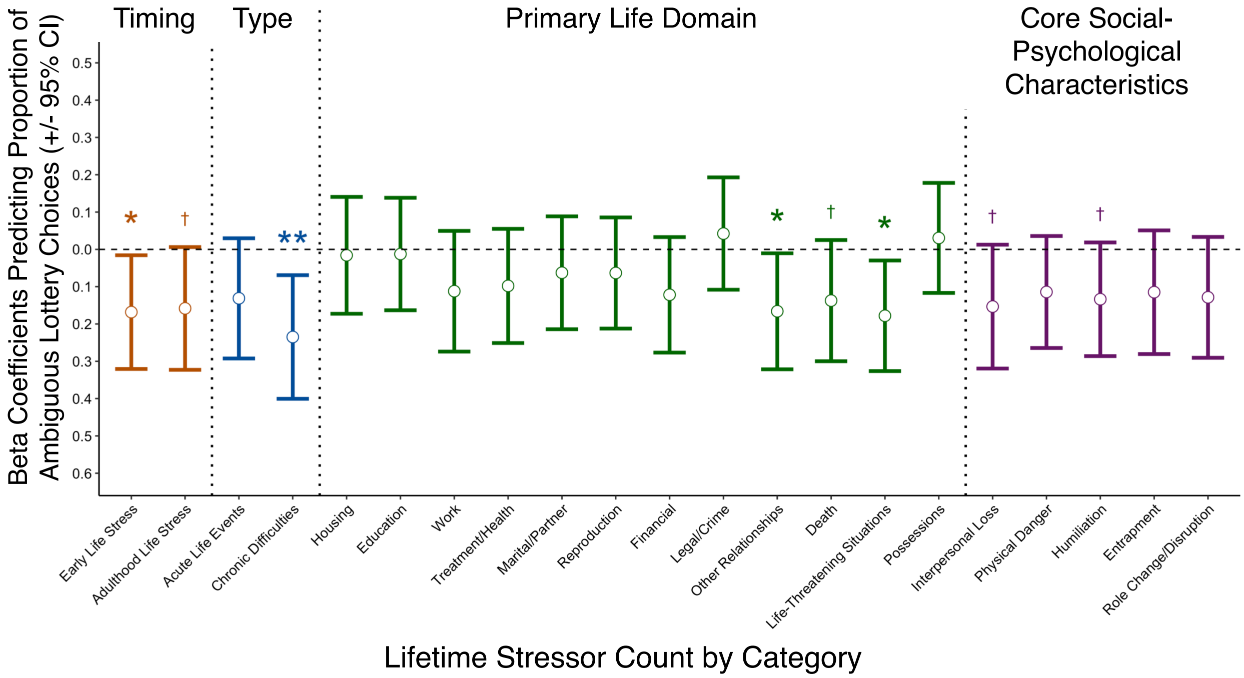

Supplementary Figure 4. Stressor-specific effects on ambiguous lottery choice (Study 2, *n*=188). Standardized beta coefficients derived from linear regressions depicting the relation between lifetime stressor count and proportion of (risk-corrected) ambiguous lottery choices controlling for age, gender, income, IQ and mental health status. Stressors on the y-axis are categorized by stressor timing (early life vs. adulthood), type (acute vs. chronic), primary life domain, and core social-psychosocial characteristics. Stressors experienced in early life (p=0.031) were predictive of ambiguous choice behavior, as were chronic difficulties (p=0.009) and stressors involving Other Relationships (p=0.041) and Life-Threatening Situations (p=0.018). Tests are uncorrected for multiple comparisons. Errors bars indicate +/- 95% CI. $^*p < .05$, $^\dagger p < .10$

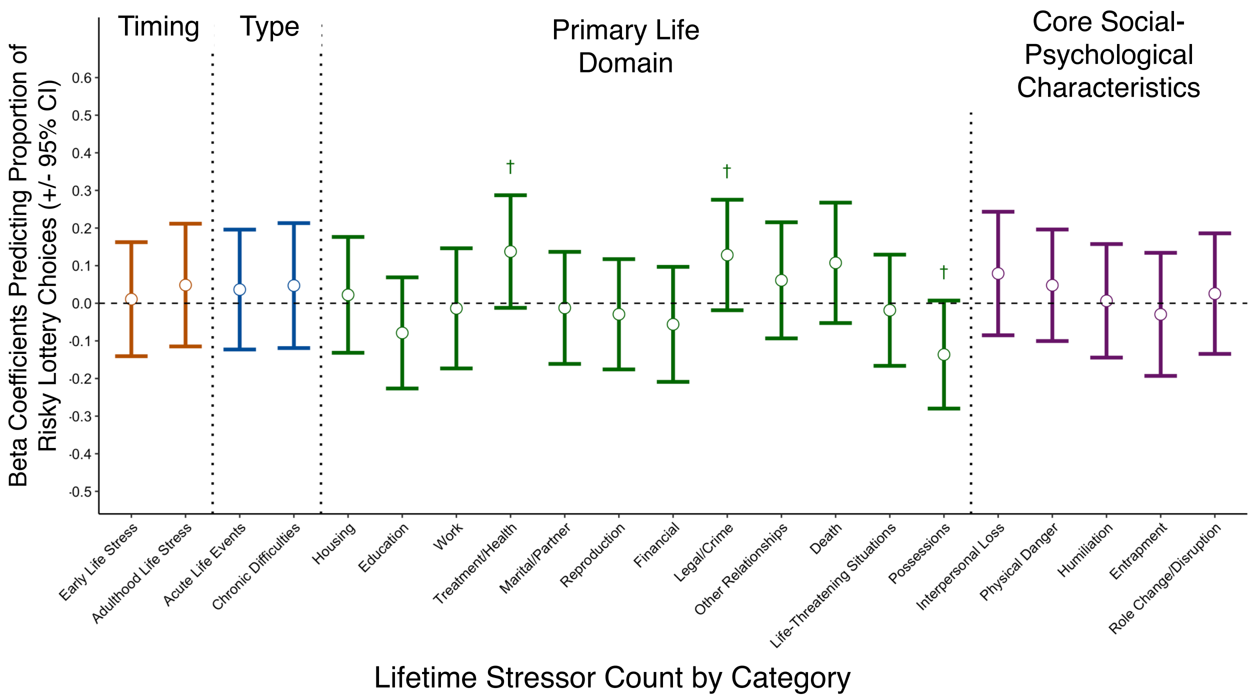

Supplementary Figure 5. Stressor-specific effects on risky lottery choice (Study 2, *n*=188). Standardized beta coefficients derived from linear regressions depicting the relation between lifetime stressor count and proportion of risky lottery choices controlling for age, gender, income, IQ and mental health status. No significant relations were observed for lifetime stressor timing, type, life domain or social-psychosocial characteristics. Tests are uncorrected for multiple comparisons. Errors bars indicate +/- 95% CI. $^*p < .05$, $^{\dagger}p < .10$

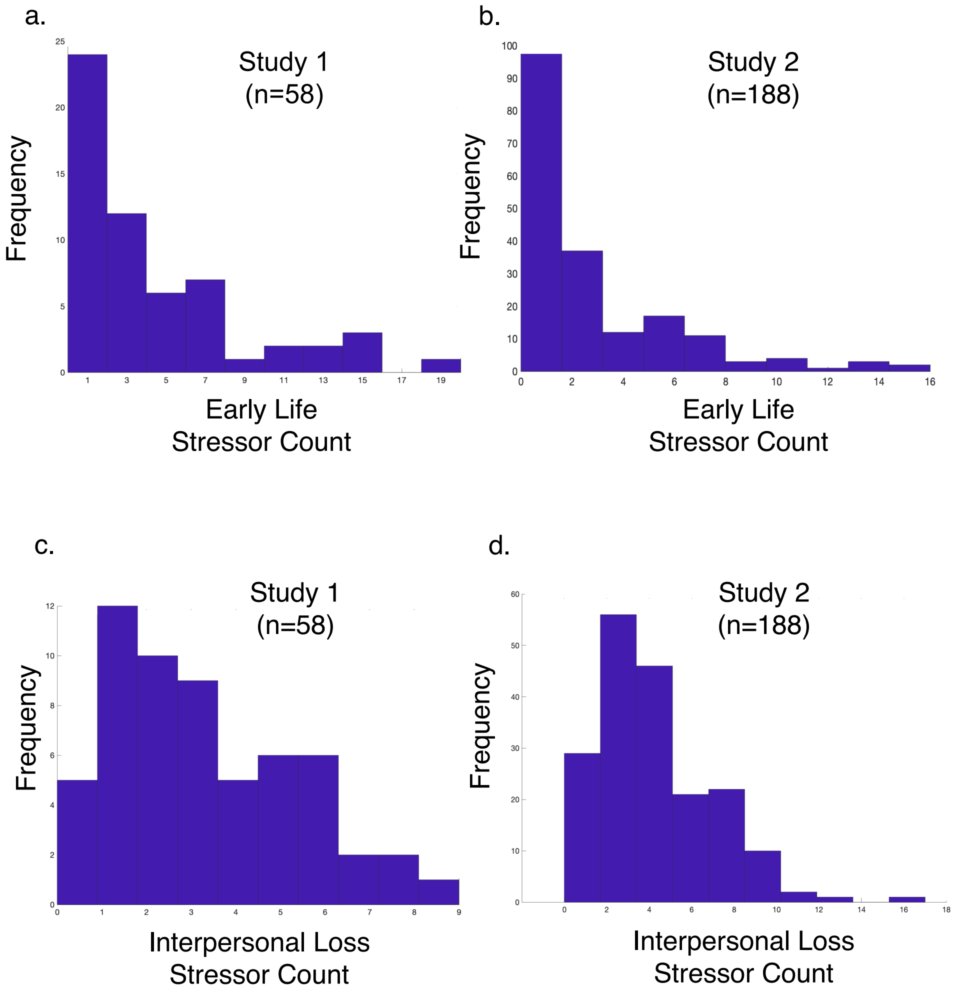

Supplementary Figure 6. Distribution data for stressors occurring early in life and for those involving interpersonal loss. Histograms depicting frequency of early life stressor count for (a) Study 1 and (b) Study 2, as well as stressors involving Interpersonal Loss for (c) Study 1 and (d) Study 2. In Study 1, participants (n = 58) exhibited a mean early life stressor count of 4.74 (*SD* = 4.81; range: 0-20) and a mean early life stressor severity of 13.15 (*SD* = 12.95; range: 0-52). In Study 2 (n = 188), the sample exhibited a mean early life stressor count of 2.71 (*SD* = 3.35; range: 0-16) and a mean early life stressor severity of 7.79 (*SD* = 9.22; range: 0-40). Participants in Study 1 and Study 2 reported a mean interpersonal loss count of 3.17 (*SD* = 2.30; range: 0-9) and 4.22 (*SD* = 2.91; range: 0-17), respectively.
