# Supplementary material for: Cumulative lifetime stressor exposure assessed by the STRAIN predicts economic ambiguity aversion
Source: Nat Commun. 2022 Mar 30;13:1686. doi: 10.1038/s41467-022-28530-2 (PMC8967930; doi:10.1038/s41467-022-28530-2)
Supplement: Supplementary file 2 — Reporting summary [file 41467_2022_28530_MOESM2_ESM.pdf]

nature research

Corresponding author(s): Dr. Candace M. Raio

Last updated by author(s): Novemebr 15, 2021

Reporting Summary

Nature Research wishes to improve the reproducibility of the work that we publish. This form provides structure for consistency and transparency in reporting. For further information on Nature Research policies, see our Editorial Policies and the Editorial Policy Checklist.

Statistics

For all statistical analyses, confirm that the following items are present in the figure legend, table legend, main text, or Methods section.

n/a	Confirmed	
☐	☒	The exact sample size (*n*) for each experimental group/condition, given as a discrete number and unit of measurement
☐	☒	A statement on whether measurements were taken from distinct samples or whether the same sample was measured repeatedly
☐	☒	The statistical test(s) used AND whether they are one- or two-sided<br>*Only common tests should be described solely by name; describe more complex techniques in the Methods section.*
☐	☒	A description of all covariates tested
☐	☒	A description of any assumptions or corrections, such as tests of normality and adjustment for multiple comparisons
☐	☒	A full description of the statistical parameters including central tendency (e.g. means) or other basic estimates (e.g. regression coefficient) AND variation (e.g. standard deviation) or associated estimates of uncertainty (e.g. confidence intervals)
☐	☒	For null hypothesis testing, the test statistic (e.g. *F*, *t*, *r*) with confidence intervals, effect sizes, degrees of freedom and *P* value noted<br>*Give P values as exact values whenever suitable.*
☒	☐	For Bayesian analysis, information on the choice of priors and Markov chain Monte Carlo settings
☒	☐	For hierarchical and complex designs, identification of the appropriate level for tests and full reporting of outcomes
☐	☒	Estimates of effect sizes (e.g. Cohen's *d*, Pearson's *r*), indicating how they were calculated

Our web collection on statistics for biologists contains articles on many of the points above.

Software and code

Policy information about availability of computer code

Data collection	Data collection for Study 1 was completed using the MATLAB toolbox Psychtoolbox.<br>Data collection for Study 2 was completed using Amazon Mechanical Turk.
Data analysis	All statistical analyses for the behavioral data were carried out in SPSS, R and MATLAB.

For manuscripts utilizing custom algorithms or software that are central to the research but not yet described in published literature, software must be made available to editors and reviewers. We strongly encourage code deposition in a community repository (e.g. GitHub). See the Nature Research guidelines for submitting code & software for further information.

Data

Policy information about availability of data

All manuscripts must include a data availability statement. This statement should provide the following information, where applicable:

- Accession codes, unique identifiers, or web links for publicly available datasets
- A list of figures that have associated raw data
- A description of any restrictions on data availability

All behavioral data will be made public after publication (OSF: https://osf.io/qvku6/).

Field-specific reporting

April 2020

Behavioural & social sciences study design

All studies must disclose on these points even when the disclosure is negative.

Study description	This is a quantitative correlational study examining the relationship between lifetime stress and economic decision preferences.
Research sample	The participant sample consistent of healthy young adult participants. For Study 1, fifty-eight healthy young adult participants (35 women), aged 25.7 ± 7.4 years (range 18–56) participated. For Study 2, the final sample consisted of 188 adult participants (82 women) aged 39.8 ± 12.14 years (range 19–73).
Sampling strategy	We employed a convenience sampling approach. No explicit sample size calculation was performed. Sample size was based on standards in the field of human decision-making and on sample size standards from the human life stress literature.
Data collection	For Study 1, behavioral data was conducted in a laboratory using MATLAB. The experimenter was present for task instructions, practice and comprehension quiz. No experimenter was present once participant's began the decision task, nor for the stress survey task. Study 2 was conducted entirely online through Amazon M-Turk.
Timing	Study 1 data were collected through 2016 and early 2017. Study 2 data were collected throughout 2019.
Data exclusions	No participants were excluded for Study 1. For Study 2, Data from 6 participants failed to record and 18 participants were excluded for demonstrating a failure to understand task instructions based on choice errors', defined as violations of first-order stochastic dominance' in greater than 15% of choices. We applied this criterion due to online nature of the task (M-turk) since such violations could indicate a failure to understand the structure of the choice task.
Non-participation	No participants dropped out/declined participation.
Randomization	Both studies were correlational in nature so no formal experimental groups were assigned.

Reporting for specific materials, systems and methods

We require information from authors about some types of materials, experimental systems and methods used in many studies. Here, indicate whether each material, system or method listed is relevant to your study. If you are not sure if a list item applies to your research, read the appropriate section before selecting a response.

Materials & experimental systems

n/a	Involved in the study	
☒	☐	Antibodies
☒	☐	Eukaryotic cell lines
☒	☐	Palaeontology and archaeology
☒	☐	Animals and other organisms
☐	☒	Human research participants
☒	☐	Clinical data
☒	☐	Dual use research of concern

Methods

n/a	Involved in the study	
☒	☐	ChIP-seq
☒	☐	Flow cytometry
☒	☐	MRI-based neuroimaging

Human research participants

Policy information about studies involving human research participants

Population characteristics	The participant sample consistent of healthy young adult participants. For Study 1, fifty-eight healthy young adult participants (35 women), aged 25.7 ± 7.4 years (range 18–56) participated. For Study 2, the final sample consisted of 188 adult participants (82 women) aged 39.8 ± 12.14 years (range 19–73).
Recruitment	For Study 1, participants were recruited using flyers posted on and around the New York University (NYU) campus, as well as electronic advertisements on NYU's Department of Psychology website. For Study 2, participants were recruited on Amazon Mechanical Turk.
Ethics oversight	Study 1: New York University Committee on Activities Involving Human Subjects. Study 2: New York University Langone Health Institutional Review Board.

Note that full information on the approval of the study protocol must also be provided in the manuscript.
